# An enigmatic presentation of *Escherichia coli* endocarditis: Emphasizing the role of brain magnetic resonance imaging

**DOI:** 10.1002/ccr3.7878

**Published:** 2023-09-11

**Authors:** Juthipong Benjanuwattra, Amanda L. Bell, Mingxiao V. Yang, Barbara L. Mora, Leigh Ann Jenkins, Pooja Sethi

**Affiliations:** ^1^ Department of Internal Medicine Texas Tech University Health Sciences Center Lubbock Texas USA; ^2^ School of Medicine Texas Tech University Health Sciences Center Lubbock Texas USA; ^3^ Division of Cardiology Texas Tech University Health Sciences Center Lubbock Texas USA

**Keywords:** emboli, endocarditis, Escherichia coli, magnetic resonance imaging, valvular heart disease, vegetation

## Abstract

**Key Clinical Message:**

Infective endocarditis should be considered in any febrile individual with acute onset neurological symptoms. If suspicion is high, a negative brain computed tomography does not virtually exclude embolism, and magnetic resonance imaging is warranted.

**Abstract:**

A diagnosis of infective endocarditis (IE) is often delayed, particularly in those infected with unusual organisms. Hereby, we report a case of a female patient presented with dysarthria, confusion, and altered mental status after being treated for *Escherichia coli* bacteremia. Computed tomography of the brain was unrevealing; however, scattered embolic phenomena were visualized on magnetic resonance imaging (MRI). The case underscores the importance of clinical awareness, particularly in the setting of unusual microorganisms, and the role of brain MRI in the diagnosis of IE.

## Graphical abstract

Brain MRI T2‐FLAIR and DWI showing multiple scattered hyperintense lesions along gray‐white junction bilaterally.

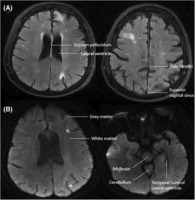

## 1 BACKGROUND

*Escherichia coli* is among the most common culprits of septicemia; however, infective endocarditis (IE) due to *E. coli* is a rare infection associated with significant mortality.^1^ Neurological manifestations are not uncommon. Although computed tomography (CT) of the brain can be promptly performed to exclude large hemorrhage,^2^ magnetic resonance imaging (MRI) exhibits greater sensitivity in embolic phenomena as demonstrated in our case.

## 2 CASE PRESENTATION

A 60‐year‐old female presented with a 9‐day history of fever, chills, dyspnea, myalgia, visual hallucination, and alteration of consciousness. She had a past medical history of multivessel coronary artery disease treated with bypass grafting, heart failure with preserved ejection fraction, pacemaker implantation for complete heart block, chronic hypoxemic respiratory failure, diabetes, and chronic kidney disease stage III.

Laboratory workup showed neutrophilic leukocytosis (WBC 16,700 cells/mm^3^, neutrophil 82.7%) and acute kidney injury (blood urea nitrogen 42 mg/dL, creatinine 2.4 mg/dL). Urinalysis showed significant pyuria with leukocyte clumps, concerning for urinary tract infection. There was no bacterial growth from the urine culture as the sample was obtained 8 h after antibiotic initiation. The first blood cultures were positive for pan‐sensitive Escherichia coli. Initially, she was treated with cefepime 2 grams IV every 12 h for 4 days. Repeat blood cultures became negative, after which were switched to trimethoprim/sulfamethoxazole 800/160 mg orally twice daily. However, she was brought back the following day with confusion, emotional lability, and dysarthria.

**FIGURE 1:**
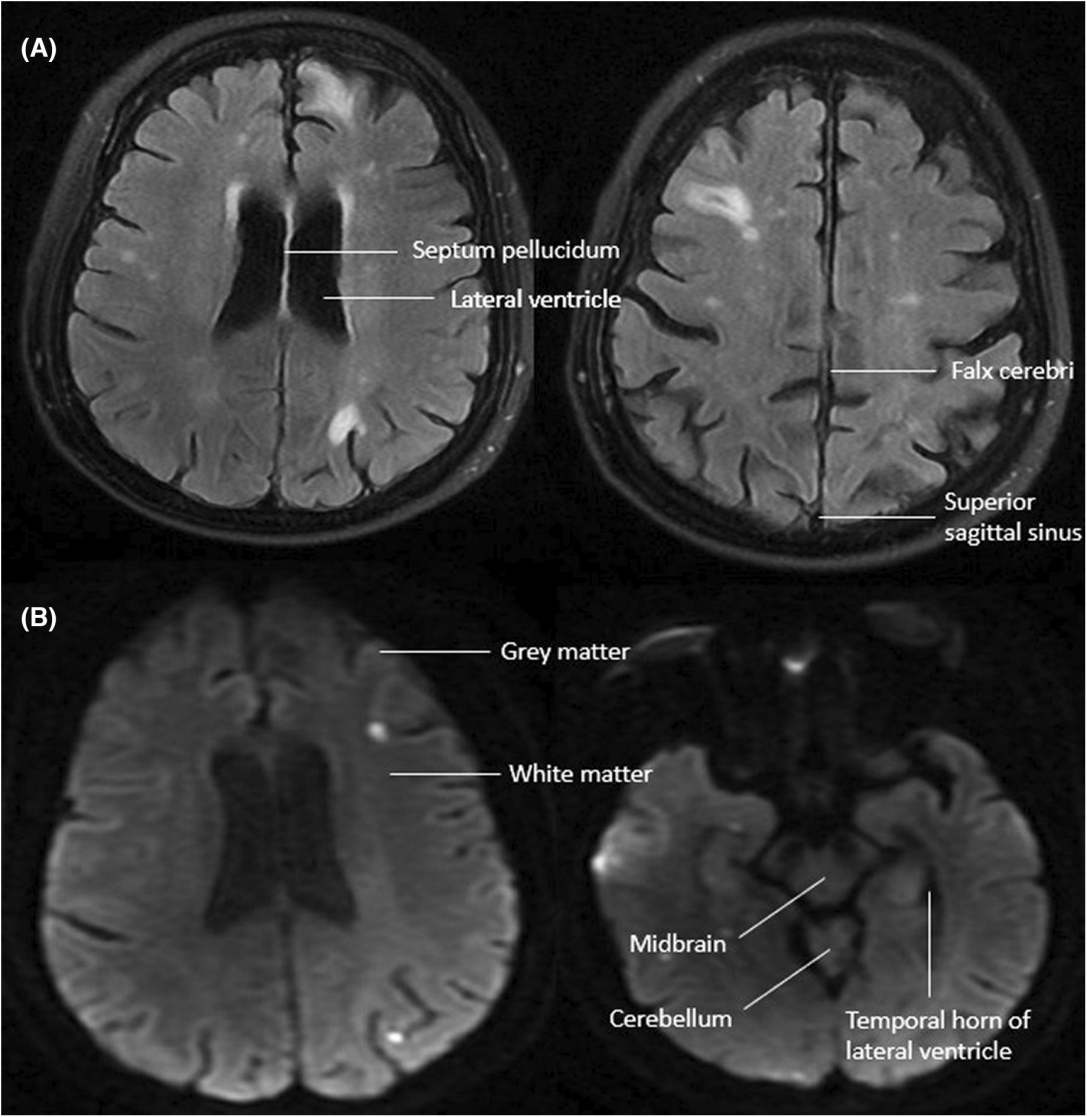
Brain MRI T2‐FLAIR (A) and diffusion‐weighted imaging (B) showing multiple punctate scattered foci of abnormal diffusion signal particularly distributed along gray‐white junction bilaterally.

**FIGURE 2:**
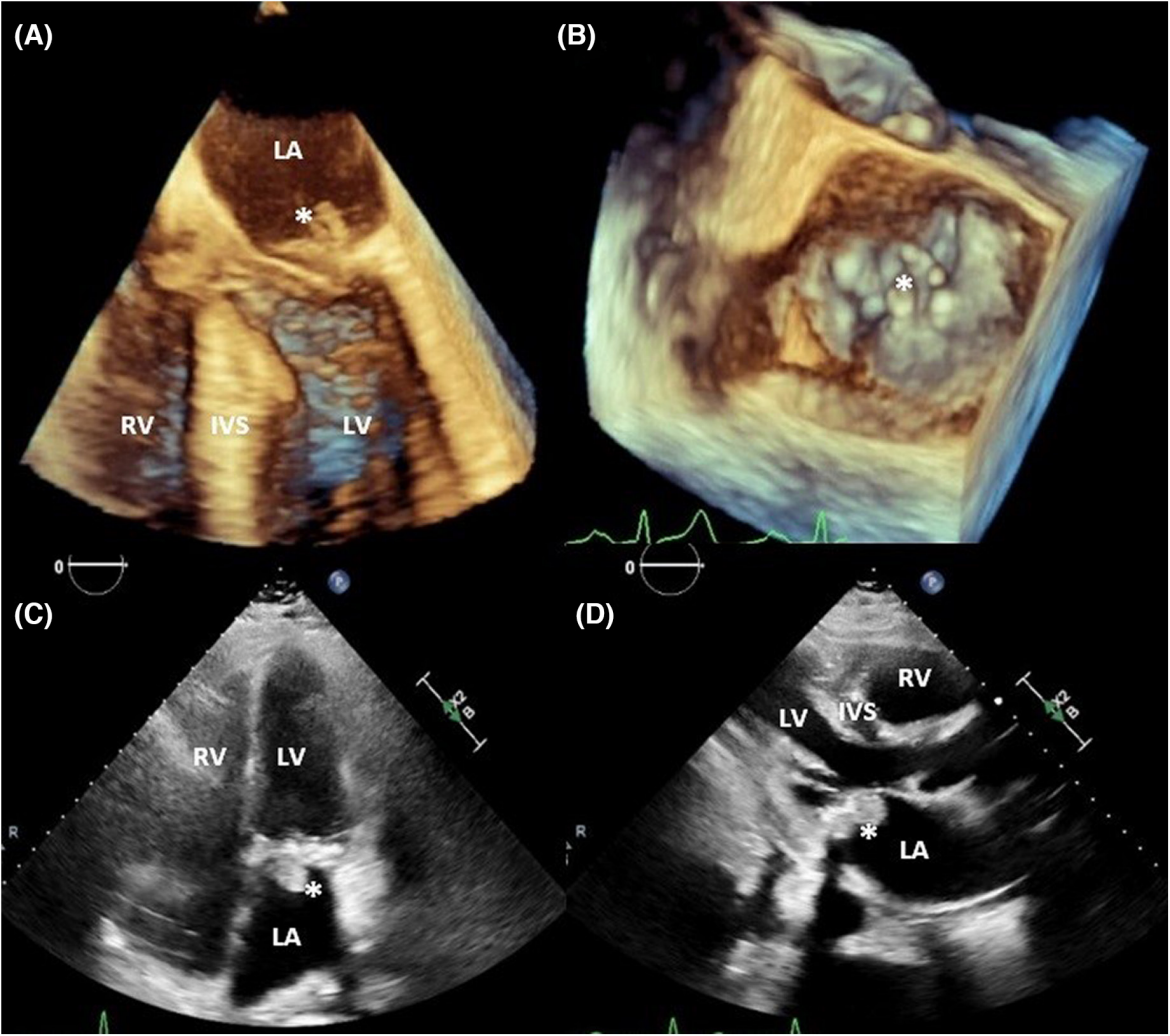
Three‐dimensional transesophageal echocardiography showing mobile serpiginous vegetations on the atrial aspect of both anterior and posterior mitral leaflets (A, B). Follow‐up transthoracic echocardiography showing calcified vegetation measuring 2 × 1.5 cm at the tip of posterior mitral leaflet (C, D). IVS, interventricular septum; LA, left atrium; LV, left ventricle; RV, right ventricle; *vegetation.

Computed tomography of the brain and angiography did not show any evidence of acute stroke or arterial stenosis. Magnetic resonance imaging of the brain was suggestive of scattered embolic phenomena **(**Figure 1 **)**. Brain MRI T2* did not reveal any cerebral microbleeds. A definite diagnosis of IE was obtained by transesophageal echocardiography (TEE), demonstrating mobile serpiginous mass on the atrial aspect of the P2 and A2 segment of the mitral valve measuring 1.3 × 0.42 and 0.4 × 0.52 cm, respectively **(**Figure 2A,B **)**. According to the modified Duke criteria,^3^ our patient had a definite diagnosis of IE by fulfilling one major echocardiographic and four minor criteria (fever, predisposing heart condition, embolic phenomenon, and positive blood culture of non‐typical microorganism). The polymerase chain reaction and culture of the valve tissue to identify *E. coli* were not available as the surgery was not performed.

On subsequent hospitalizations, blood cultures drawn at 12 h apart were consistently negative, including antibodies to *Coxiella burnetii*. She was diagnosed with culture‐negative infective endocarditis due to preceding *E. coli* septicemia and recent antibiotic administration. Cardiothoracic surgery and infectious teams were consulted. Given the high surgical risk and absence of clinical heart failure or severe regurgitation, a prolonged course of intravenous ceftriaxone for 6 weeks was planned.

Two weeks later, she presented with worsening dyspnea, hypoxia, and renal failure, secondary to coronavirus infection. Repeat TTE continued to demonstrate calcified vegetation 2 × 1.5 cm at the tip of the posterior mitral leaflet with mobile component measuring 1.5 × 0.7 cm **(**Figure 2C,D **)**. Ejection fraction was normal and only trace mitral regurgitation was noted. Again, blood cultures were negative. Ceftriaxone was escalated to cefepime and daptomycin for another 6 weeks. Unfortunately, she was hospitalized again a few days prior to completing antibiotics with sudden onset leg weakness and fall. Repeat brain MRI showed multiple new lesions in juxtacortical left temporal lobe, right cerebellum, and right periventricular white matter. She was evaluated again for the cardiac surgery. In light of overall condition and severely impaired lung function test, a decision was made by the family to pursue hospice care.

## 3 DISCUSSION

Infective endocarditis is an uncommon life‐threatening infection with an annual incidence of up to 10 per 100,000 of the population.^4^ Based on a population‐based study of 497 patients, *Staphylococcus aureus* accounted for 26.6% of the cases, followed by *Viridans* streptococci at 18.7%, group D streptococci at 12.5%, and enterococci at 10.5%.^5^ The majority of gram‐negative organisms implicated as the main bacterial sources of IE were of the Haemophilus, Aggregatibacter, Cardiobacterium, Eikenella, and Kingella (HACEK) groups.^6^

Despite being previously attributed to intravenous drug use, most non‐HACEK endocarditis cases are more likely to be related to healthcare contact as well as implanted cardiac devices such as prostheses and defibrillators, although the absolute risk is still trivial.^7^ In addition to a major concern of an increasing prevalence of drug‐resistant gram‐negative bacilli, non‐HACEK endocarditis is associated with notable rates of intracardiac abscess, conduction abnormality, and mortality.^7^ Among the non‐HACEK counterparts, *Enterobacteriaceae* account for less than 1% of IE cases.^5^ Though *E. Coli* is a common source of sepsis, it is a much less common cause of IE, accounting for only 0.5%.^1^ Genitourinary and gastrointestinal tracts are presumed to be frequent sources of bacterial acquisition with *E. Coli* being the most common culprit followed by *Pseudomonas aeruginosa*.^7^ *Escherichia coli* with phylogenetic type B2, a pathogenic lineage associated with extraintestinal infection, was isolated from an 80‐year‐old woman with emphysematous endocarditis, raising a possibility of certain virulence factors that could have predisposed to endocardial invasion.^8^ Several comorbidities are reportedly associated with *E. coli* endocarditis including end‐stage renal disease, diabetes, malignancies, and chronic alcohol consumption.^1^

Although there is a sevenfold increase in 30‐day mortality if infected leads are retained, removal of implantable devices carries its own risk including cardiac tamponade, stroke, vascular and cardiac injury.^9^ Therefore, the decision pertaining to device removal should be individualized, mostly in the setting of confirmed device infection such as pocket infection, lead vegetation, or persistent bacteriemia without an alternative source. Microorganisms should also be taken into consideration, as device removal is recommended in Staphylococcus, Propionibacterium, or Candida infection, while antibiotic therapy is usually adequate in gram‐negative bacterial infection.^9^

Although the incidence of neurological manifestations associated with IE varies among studies ranging from 30% to 47%, obtaining a diagnosis is frequently delayed, resulting in increased morbidity and mortality.^10^, ^11^, ^12^ Neuropsychiatric symptoms including confusion, personality change, and hallucinations were reported in up to 50% of patients, with the highest prevalence noted among the elderly.^11^ The patient's neurological exam was mostly non‐focal, in which moderate dysarthria and confusion were the initial presentation. Her embolic location might have explained the dysarthria, while confusion was due to a combination of embolic stroke and septic encephalopathy. Although she presented with fever and neurological symptoms, Lumbar puncture was not performed given that criteria for infective endocarditis were fulfilled and meningeal irritation was absent.

Central nervous system lesions involve either a single vessel, particularly the middle cerebral artery, or multiple vessels and may be complicated by purulent meningitis or brain abscess.^11^ Mycotic aneurysm and intracranial haemorrhage, which might occur months to years after the infection has resolved, are catastrophic sequela resulting from suppurative inflammation of the arterial wall.^11^

In a systemic review of patients with IE, brain MRI exhibits greater sensitivity in identifying lesions such as microinfarcts and small hemorrhagic lesions undetected by CT scan.^2^, ^12^ Asymptomatic cerebral lesions were noted on systematic cerebral MRI in 78% of cases.^13^ Vitali et al. showed that 83% of cerebral lesions were detected on MRI as opposed to only 15% detected on CT.^2^ Using systematic brain MRI, vegetations larger than 4 mm in length correlated with cerebral embolism and most small lesions were, not surprisingly, undetectable on CT scan.^13^ The early use of brain MRI with angiography led to a change in diagnostic classification from non‐definite IE to either definite or possible IE according to modified Duke criteria in 17 of 53 patients.^14^ Using both DWI and FLAIR imaging, the age of lesions can be determined. Acute lesions are detected in DWI with subtle changes in FLAIR imaging, while subacute and chronic lesions will be shown as hyperintensity, with or without leukomalacia, in FLAIR protocol but not in DWI.^15^ The initial MRI results in our patient, therefore, were suggestive of acute embolic stroke.

Although the role of brain imaging, either by CT or MRI, is solid in patients with an established diagnosis of IE and neurological symptoms, the role of brain MRI as a diagnostic modality has not been implemented and a decision should be individualized on a case‐by‐case basis. Although urgent surgery is indicated in certain conditions including but not limited to persistent bacteremia despite appropriate antibiotic treatment, hemodynamic instability due to valvular insufficiency, and large vegetation size, the timing for surgery in those with preoperative neurological complications remains ambiguous due to a concern of hemorrhagic transformation.^16^ A gap of knowledge as to whether an expedited surgical intervention confers any clinical benefits in patients whose a diagnosis of IE with embolic strokes is early made by brain MRI remains to be clarified.

## 4 CONCLUSION

A diagnosis of IE is often delayed or missed, particularly in the setting of bacteremia caused by an unusual organism. *E. coli* has been increasingly reported as a culprit in susceptible patients. In those with neurological symptoms, a negative brain CT does not reliably exclude embolic phenomena and brain MRI should be considered if clinical suspicion remains high.

## AUTHOR CONTRIBUTIONS

**Juthipong Benjanuwattra:** Conceptualization; writing – original draft; writing – review and editing. **Mingxiao V. Yang:** Writing – original draft. **Amanda L Bell:** Writing – original draft. **Barbara L Mora:** Writing – original draft. **Leigh Ann Jenkins:** Supervision; validation; writing – review and editing. **Pooja Sethi:** Conceptualization; investigation; supervision; validation.

## CONFLICT OF INTEREST STATEMENT

The authors declare that they have no competing interests.

## CONSENT STATEMENT

Written informed consent was obtained from the patient to publish this report in accordance with the journal's patient consent policy.

## Data Availability

Not applicable.
